# The Outcome of Placing the Medial K-wire First and Then the Lateral K-wire in Treating Supracondylar Humerus Fractures in Children Treated by Closed Reduction

**DOI:** 10.7759/cureus.30911

**Published:** 2022-10-31

**Authors:** Tej P Gupta, Sanjay K Rai, Amit Kale, Deepak C Reddy

**Affiliations:** 1 Orthopaedics, Base Hospital Delhi Cantt, Delhi, IND; 2 Orthopaedics, Military Hospital Ambala Cantt, Ambala, IND; 3 Orthopaedics, Base Hospital, Guwahati, IND; 4 Radiology, Military Hospital Ambala Cantt, Ambala, IND

**Keywords:** closed reduction, lateral pinning, supracondylar fracture humerus, percutaneous pinning, medial wire placement

## Abstract

Background

Displaced Gartland type III and IV supracondylar fractures are difficult to reduce and invariably require closed pining. After closed reduction, taking the anteroposterior (AP) view does not present any problem but when the elbow is placed in flexion and the limb is rotated internally to take a lateral view, the reduction is invariably lost. However, the reduction stays when the arm is rotated outwards, keeping the medial condyle up. This stimulates the idea of whether the medial pin can be placed first and then the two lateral pins to stabilize the fracture.

It is very frustrating for young orthopedic surgeons to see reduction getting lost during internal rotation after first doing lateral pinning. There is no clear guideline on which side should be fixed first.

Hypothesis

We hypothesized that placing the medial pin first maintains the reduction and facilitates the subsequent placing of lateral pins without the loss of reduction.

Materials and methods

A total of 170 children with displaced supracondylar humerus fractures were included in the study. A total of 120 children were grouped in the medial wire first group, and 50 were placed in the lateral wire first group, which was the control group. The mean age of the children was 7.5 years (range 2-13 years). The gender ratio (M: F) was 5:3; the left elbow was involved in 68% of the injuries, whereas the right elbow was involved in 32% of the injuries. All 170 children had an extension-type injury, with 91 (53.5%) fractures being Gartland type III and 79 (46.45%) fractures being type IV.

Results

Results were recorded as per Flynn's criteria. At the end of two years of follow-up, the children in the medial wire first group 117 (97.5%) showed excellent results and three (2.5%) children showed good results, whereas, in the lateral wire first group, 48 (96%) children showed excellent results and two (3.8%) children showed good results. There was a significant difference in the mean surgical time of 20.11±15.43 minutes in the medial wire first group vs 41.23±19.65 minutes in the lateral first group (p = 0.0021). None of the children developed permanent ulnar nerve palsy.

Conclusions

Placing the medial K-wire first rather than the conventional placing of the lateral wire first helps in maintaining the reduction and allows for the subsequent placement of lateral K-wires without losing the reduction, thus minimizing fixation time and producing good results.

## Introduction

A supracondylar fracture of the humerus is the most common fracture in children [1]. Grossly displaced supracondylar fractures are very difficult to reduce by close reduction, as it is difficult to maintain the reduction, and sometimes there is the involvement of neurovascular structures [2-4]. There is no consensus on the treatment of this fracture available in the literature [5,6]. In grossly displaced extension-type supracondylar fractures, controversy remains regarding the configuration of the pin fixation. A cross-pin configuration is believed to be mechanically more stable than lateral pins alone as noted by many authors [7,8]. However, the use of a medial pin can injure the ulnar nerve [9,10].

Tachdjian's Pediatric Orthopaedics by Herring JA describes safe medial pin insertion techniques to minimize iatrogenic ulnar nerve injury, including ‘displacing’ the soft tissue, especially posteriorly, by using the thumb to protect the ulnar nerve and positioning the elbow in extension for medial pin insertion as mentioned [11]. We also followed these steps while passing the medial pin. Edmonds et al. and Woo et al. followed the steps given in Tachdjian's Pediatric Orthopaedics textbook by Herring JA regarding safe pin holding, the position of the elbow, and insertion of a medial pin in order to minimize ulnar nerve injury [12,13]. In the year 2018, Woo et al. treated 125 children with supracondylar fractures by medial pin fixation first with good results [13]. There is no study in the literature regarding which K-wire should be placed first so that the reduction stays during fracture fixation with minimal anesthesia time.

This study's objective was to assess the placement of the medial K-wire first rather than the conventional placing of the lateral pin first and to assess the surgical time, anesthesia time, and which method makes fixation easier and minimizes the chances of displacement of the distal fragment during fixation.

## Materials and methods

The present study was a prospective interventional study that included 170 children admitted with displaced supracondylar fractures (Gartland types III and IV). The study was carried out at three different centers by the same orthopedic surgeons between August 2013 and July 2019, with a two-year follow-up included, after institutional medical ethics committee approval (151BH/Ortho/ 08). Written informed consent was obtained from all the parents.

A total of 193 children were received during the study period. Only 170 children qualified to be included in the study, as we could achieve closed reduction in all of them. In the rest of the children, i.e. in 23, we could not achieve closed reduction, and, subsequently, open reduction was done, and they were therefore excluded from the study. As we have not included those children who required open reduction because it was out of the scope of the study. Finally, 120 children were grouped in the medial wire first group, and 50 were placed in the lateral wire first group, which was the control group.

Inclusion criteria were ages between 2 and 13 years and displaced supracondylar fractures (Gartland types III and IV). Exclusion criteria were undisplaced supracondylar fractures, displaced supracondylar fractures with vascular compromise, and open displaced supracondylar fractures.

After pre-anaesthesia check-ups, children were taken for closed reduction and pinning under sedation. After the sensitivity test, a single dose of intravenous cefotaxime was given on induction of anesthesia, according to the child's weight. The child was placed in a supine position with the shoulder at the edge of the image intensifier-compatible tableside support. The injured elbow, arm, and forearm were prepared and draped, leaving the lower third of the arm, elbow, and forearm exposed up to the wrist so that the radial pulse could be easily felt during manipulation and during surgery. A sterile drape-covered image intensifier was placed by the side of the table and longitudinal traction was applied with the elbow in 15-20-degree flexion keeping the forearm in supination along with countertraction. Medial or lateral displacement was corrected while continuing traction and counter-traction by lateral or medial force. We could achieve varus angulation correction by pronating the forearm and subsequently, flexing the elbow and applying pressure on the distal fragment and on the olecranon using the surgeon’s thumb in order to push the distal fragment anteriorly.

**Figure 1:**
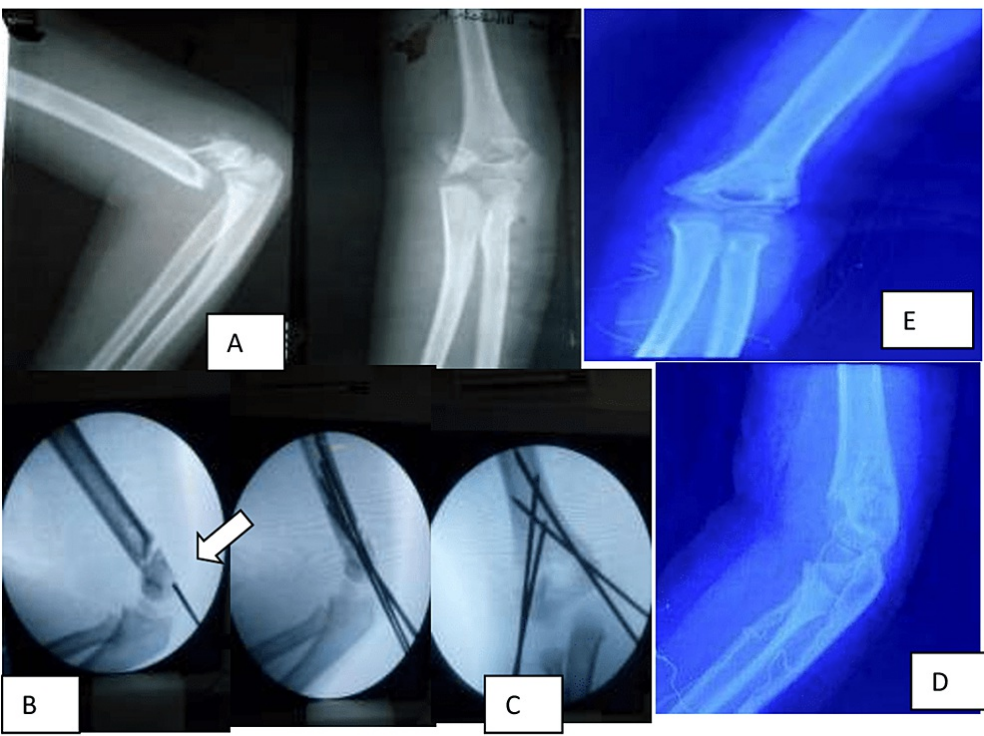
Preoperative X-ray anteroposterior and lateral views showing a Gartland type III supracondylar fracture (A) Preoperative X-ray, (B) Putting the medial wire first as shown by arrow, (C) postoperative x-ray, (D&E) At three weeks following wire removal

**Figure 2:**
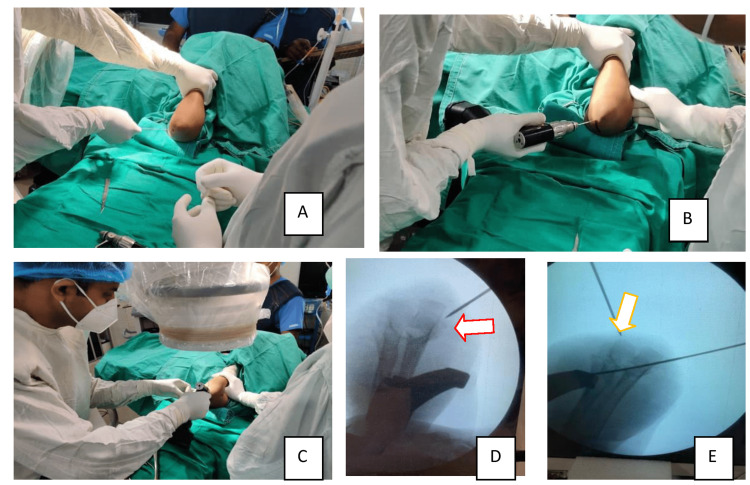
Intraoperative placement of the medial pin first Placing the medial K-wire in progress (A) Localization of the medial epicondyle with a K-wire without mounting on a drill, (B&C) Putting the medial wire first, (D) Position of the medial K-wire confirmed in the C-arm image as shown by the arrow, (E) Position of the lateral K-wire confirmed in the C-arm image as shown by the arrow

A post-reduction anteroposterior (AP) view through the fully flexed elbow and a medial view (medial condyle upward, and not the lateral condyle) were taken by the external rotation of the shoulder, keeping the elbow flexed more than 100°, as shown in Figure 1. After reduction, the radial pulse was checked. In the AP view, when the Baumann angle was restored, the reduction was considered acceptable, along with the alignment of medial and lateral columns, and in the lateral view, when the anterior humeral line passed through the capitellum. A 1.6-1.8 mm smooth K-wire was used for the maintenance of reduction. In 120 cases, we placed the medial K-wire first and then the lateral wire (Figure 1). The technique of placement of the medial K-wire was to first place the tip of the wire on the palpably most prominent medial epicondyle. This was confirmed under the C-arm. We took precautions while passing the medial pin so that it did not slip posteriorly by placing the surgeon's opposite thumb just behind the medial epicondyle. After confirmation, the medial wire was advanced till it just crossed the opposite lateral cortex; the position was confirmed by gently extending the elbow in the AP view. After confirmation, the medial wire was advanced till it just crossed the opposite lateral cortex; the position was confirmed by gently extending the elbow in the AP view. After confirmation, the elbow was again flexed maximally and the shoulder internally rotated so that the lateral epicondyle was placed upwards and the lateral K-wire was placed as shown in Figure 2.

After confirmation of the position of the initial first medial and lateral pin, the second medial and lateral pin was inserted in a similar fashion. Finally, the reduction, as well as the position of all four K-wires (two medial and two lateral) in the AP and lateral views, were confirmed.

Finally, the reduction, as well as the position of K-wires in the AP and lateral views, were confirmed. After confirmation of radial pulse, the wire was bent and cut outside the skin and a sterile dressing was applied. An above-elbow posterior plaster slab in 90° elbow flexion and the mid-prone position of the forearm was applied. Most of the children were discharged the next day with oral antibiotics and oral analgesics after checking X-rays. At three weeks postoperatively, the plaster was removed and X-rays were taken for evidence of callus formation. The K-wires were taken out once radiological and clinical signs of the union were detected in all age groups of children. If the callus was not formed, a plaster slab was reapplied for another week (till 4 weeks postoperatively). We had to retain K-wires till four weeks postoperatively in 23 children, due to lack of clinical union at three weeks. There was no case where the wires were to be retained for more than four weeks. Initially, for two weeks, all the children were encouraged to do supervised intermittent active elbow flexion and extension exercises. At eight weeks, a radiological examination was done to assess the union. All the children were advised flexion, extension, supination, and pronation exercises of the elbow till the final follow-up in the OPD at 12 and 24 weeks when they were evaluated, and the results were noted according to the criteria described by Flynn et al. [14] (which includes a change in carrying angle and loss of movement).

The results so obtained were further categorized according to variables such as time elapsed between injury and surgical fixation, grade of fracture, age of the child, and statistical analyses of these results using the chi-square test as a test of significance.

Statistical analysis

We used SSPS ver. 19 (IBM Corporation, Armonk, NY) and Microsoft Office Excel 2019 (Microsoft Corporation, Redmond, WA) for analysis. For the statistical test of significance, the chi-square test was used to compare the results among different variables.

## Results

Among the 170 children in our study (mean age 7.5 years; range 2-13 years), 141 (82.9%) were boys and 29 (17%) were girls. The M: F ratio was approximately 4:1, and the left elbow was involved in 68% of the injuries, whereas the right elbow was involved in 32% of the injuries. The most common mode of trauma was falling from a height with the elbow in extension. All 170 children had an extension-type injury, with 91 (53.5%) fractures being Gartland type III and 79 (46.45%) fractures being type IV. We noted posteromedial displacement in 63% of fractures, whereas posterolateral displacement was noted in 37% of fractures.

We noted fewer preoperative complications, including feeble radial pulse in 21 children and radial nerve palsy in 4 (4.8%) cases. A total of 133 (78.2%) children were operated on within 12 hours of injury, 12 (7%) within 12-24 hours, 22 (12.9%) within 24-48 hours, and 03 (1.7%) were operated on after 48 hours of injury because of late presentation and due to massive swelling at the elbow. The maximum percentage of excellent results was noted in the group operated within 12 hours of injury in 113 children (84.9%), and the highest percentage of poor results in 22 children (12.9%) was observed in those operated within 24-48 hours after injury (Table 1).

**Table 1:** Results according to the time elapsed between injury and surgery in both groups

	Results				
Time between injury and surgery	Excellent	Good	Fair	Poor	Number of children
< 12 hr	113(84.9%)	09(6.7%)	08(6%)	03(2.2%)	133(78.2%)
12- 24 hr	07(5.8%)	02(1.6%)	03(2.5%)	0	12(7%)
24-48 hr	06(5%)	04(3.3%)	05(4.1%)	07(5.8%)	22(12.9%)
48-72 hr	0	0	02(1.6%)	01(0.8%)	03(1.7%)
Total	126(74.11%)	15(8.8%)	18(10.5%)	11(6.4%)	170

The chi-square test was used to analyze data and for significance, and we noted that the duration between injury and surgery has no statistical significance. (χ2 = 12.791; df = 12; P > 0.05).

The maximum percentage of excellent results 69 (72.6%) was achieved in the age group of two to six years. However, after applying the chi-square test, there was no statistically significant difference in results between different age groups (x2 = 6.983; df = 6; P > 0.05), as shown in Table 2.

**Table 2:** Results according to the age group of children at the time of presentation

Age in years	Results				
	Excellent	Good	Fair	Poor	Number of children
2-6	69 (72.6%)	12(12.6%)	9(9.4%)	05(5.2%)	95
7-10	54(85.7%)	01(1%)	5(7.9%)	03(4.7%)	63
11-13	03(25%)	02(16.6%)	4(33.3%)	03(25%)	12
Total	126(74.11%)	15(8.8%)	18(10.5%)	11(6.4%)	170

The maximum percentage of excellent results in 69 children (75.8%) was noted in Gartland grade III type fractures. However, it was 57 (72.1%) in type IV fractures, as shown in Table 3.

**Table 3:** Results according to the types of fractures in both groups

Gartland type/ Grade	Results				
	Excellent	Good	Fair	Poor	Number of children
Grade II	69(75.8%)	09(9.8%)	11(12%)	02(2.1%)	91(53.5%)
Grade III	57(72.1%)	06(7.5%)	07(8.8%)	09(11.3%)	79(46.45%)
Total	141(74.11%)	15(8.8%)	18(10.5%)	11(6.4%)	170

The loss of range of flexion, extension, and loss in carrying angle in both the groups was not significant (p=0.431, 0.632, and 0.712, respectively). However, the mean time of surgery and anesthesia was significantly less in the medial first group as compared to the lateral first group (p= 0.0021 and 0.0431 respectively) (Table 4).

**Table 4:** Analysis of loss of flexion, extension, carrying angle, surgery time, and anesthesia time in both groups Footnote: * mean surgical time – starts after achieving closed reduction and placement of all four pins (two medial and two lateral pins), time taken in the application of plaster is not included in surgical time. ** Anesthesia time – starts from induction until recovery

Parameters	Medial first group	Lateral first group	P-value (student’s t-test)
Mean loss of flexion	7.21±5.23	7.34±5.33	0.431
Mean loss of extension	2.54±4.19	3.65±4.43	0.632
Mean change in Carrying angle	6.23±2.45	7.41±3.12	0.712
Mean time of surgery (in minutes) *	20.11±15.43	41.23±19.65	0.0021
Anesthesia time ** (in minutes)	36.22 ±7.55	45.18 ±23.37	0.0431

We recorded pin tract infection in 03 (1.76%) cases after seven days postoperative, and loss of reduction in 03 (1.76%) children detected on the day of discharge as an early postoperative complication. All three cases of loss of reduction were further treated by re-reduction and placement of new K-wires on the next day. The reason for the loss of reduction was due to the loosening of the pin that no more held the fracture.

In the present study, we did not find any significant differences in the rate of complication in either group. However, two children developed ulnar nerve palsy in the medial first group, which was recovered in the next six months follow-up without any intervention (Table 5).

**Table 5:** Early complications within four weeks post-surgery

Nature of complication	Medial first group (n=120)	Lateral first group ( n=50)	P-value (student’s t-test)
Pin tract infection	01	2	0.612
Pin loosening	03	1	0.784
Pin migration	-	1	0.654
Loss of reduction	01	2	0.823
Ulnar nerve palsy	02	-	0.431
Myositis ossificans formation	-	2	0.321
Total	7	8	0.463

At the final follow-up at two years, none of the children developed elbow stiffness; however, three children (2.5%) in the medial first group and two (4%) children in the lateral first group developed cubitus varus deformity (Table 6).

**Table 6:** Outcome at six months, one year, and two years post-surgery

Follow-up period	Myositis ossificans formation	Ulnar nerve palsy	ROM 30-100	Cubitus varus ( CA -5 to 10 degrees)
At 6 months follow-up				
Medial first group	02	-	5	Could not be measured due to FFD
Lateral first group	1	-	3	Could not be measured due to FFD
At one-year follow-up				
Medial first group	1	-	-	3
Lateral first group	1	-	-	5
At two-year follow-up				
Medial first group		-	-	3
Lateral first group		-	-	2

Functional limitation of the elbow range of motion compared with the uninjured side was measured using a goniometer at the final follow-up at two years (Table 7). All patients were also evaluated with the Flynn rating scale at follow-up.

**Table 7:** Flynn criteria for grading supracondylar fractures at the final two years follow-up

Rating	Loss of motion			Loss of Carrying Angle		
		Medial first	Lateral first		Medial first	Lateral first
Excellent	0-5	117(97.5%)	48(96%)	0-5	117	48
Good	5-10	3(2.5%)	2(3.8%)	5-10	3	2
Fair	10-15	-	-	10-15	-	-
Poor	>15	-	-	>15	-	

## Discussion

The cross K-wire configuration is commonly used for pediatric supracondylar humeral fracture fixation. It is superior and resists axial rotation; however, it requires the insertion of a medial pin as well. The medial pin insertion possesses a potential risk of ulnar nerve injury. In order to avoid iatrogenic ulnar nerve injury, the lateral pin-only configuration came into practice with good outcomes. Many published data have shown that the lateral-only configuration is as good as the cross K-wire configuration with regard to instability and incidence of nerve injury [15,16]. However, we believe that after the reduction, when the arm is rotated internally for a lateral view for insertion of the lateral pin first, the reduction is invariably lost. However, if a medial pin was inserted first by rotating the arm externally, keeping the medial epicondyle up, and then the arm is rotated internally to insert a lateral pin, the reduction stays. Insertion of a medial pin first also helps in fracture stabilization when medial-wall comminution is present. A medial pin is usually needed to maintain stability as postoperative instability has been noted in the literature where only two lateral pins were used.

Reynolds RA and Mirzayan R described techniques of medial pin insertion by maintaining elbow flexion and by drawing lines on the skin around the elbow [17]. Green et al. and Gordon et al. both described a small incision over the medial epicondyle for direct visualization before placing a medial pin [18,19]. Michael and Stanislas described the use of a nerve stimulator to identify the location of the ulnar nerve [20].

We used our fingers or thumb for the precise identification of the medial epicondyle. First, the K-wire tip was placed on the most prominent medial epicondyle and then the drill was mounted onto it and advanced slowly in a controlled fashion; thus, at this time, only one pin was necessary. With this technique, K-wire slippage or plunging and ulnar nerve injury were not noted. Further, we do not recommend locating the starting point on the medial epicondyle with a mounted K-wire on the drill.

Swenson proposed that once the starting point is located, insert the K-wire on an acutely flexed arm to maintain the fracture's reduction; this supports our recommendation that keeping the elbow flexed at about 100° is sufficient to insert the medial pin first [21]. We further noticed that when the elbow is flexed beyond 100°, palpation of the medial epicondyle was difficult in the presence of swelling.

We observed that iatrogenic ulnar nerve injury usually results from the incorrect insertion and placement of the K-wire because the starting point is often posterior or inferior to the medial epicondyle.

Few authors reported that the ulnar nerve may be displaced anteriorly to the medial epicondyle in about 20% of cases in children [22-24]. In the present study, we did encounter two cases of ulnar nerve palsy but they recovered fully in the next six months of follow-up.

Limitations of the present study

One limitation of this study is the short follow-up of two years only, and we did not follow up on the loss of carrying angle and the development of cubitus rectus or varus deformity after two years.

## Conclusions

In the present study, placing a medial K-wire first in type III and IV fractures holds the reduction well in place and allows the placement of lateral K-wires subsequently. The mean surgical and anesthesia time was significantly less in the medial first group. The surgical technique adopted in the present study fully satisfy the existing guidelines and recommendations to avoid implant-related or iatrogenic ulnar nerve injury at the time of medial pin insertion for supracondylar humerus fracture fixation. It is quickly learned, easily reproducible, and produces excellent results.
